# Liver and Muscle in Morbid Obesity: The Interplay of Fatty Liver and Insulin Resistance

**DOI:** 10.1371/journal.pone.0031738

**Published:** 2012-02-16

**Authors:** Mariana Verdelho Machado, Duarte M. S. Ferreira, Rui E. Castro, Ana Rita Silvestre, Teresinha Evangelista, João Coutinho, Fátima Carepa, Adília Costa, Cecília M. P. Rodrigues, Helena Cortez-Pinto

**Affiliations:** 1 Departamento de Gastrenterologia, Unidade de Nutrição e Metabolismo, Hospital Santa Maria, Faculdade de Medicina de Lisboa, IMM, Lisbon, Portugal; 2 Research Institute for Medicines and Pharmaceutical Sciences (iMed.UL), Faculty of Pharmacy, University of Lisbon, Lisbon, Portugal; 3 Departamento de Neuropatologia, Hospital Santa Maria, Lisbon, Portugal; 4 Departamento de Cirurgia, Hospital Santa Maria, Lisbon, Portugal; 5 Departamento de Anatomia Patológica, Hospital Santa Maria, Lisbon, Portugal; Copenhagen University Hospital Gentofte, Denmark

## Abstract

**Introduction:**

Nonalcoholic fatty liver disease (NAFLD) can be seen as a manifestation of overnutrition. The muscle is a central player in the adaptation to energy overload, and there is an association between fatty-muscle and -liver. We aimed to correlate muscle morphology, mitochondrial function and insulin signaling with NAFLD severity in morbid obese patients.

**Methods:**

Liver and deltoid muscle biopsies were collected during bariatric surgery in NAFLD patients. NAFLD Activity Score and Younossi's classification for nonalcoholic steatohepatitis (NASH) were applied to liver histology. Muscle evaluation included morphology studies, respiratory chain complex I to IV enzyme assays, and analysis of the insulin signaling cascade. A healthy lean control group was included for muscle morphology and mitochondrial function analyses.

**Results:**

Fifty one NAFLD patients were included of whom 43% had NASH. Intramyocellular lipids (IMCL) were associated with the presence of NASH (OR 12.5, p<0.001), progressive hepatic inflammation (p = 0.029) and fibrosis severity (p = 0.010). There was a trend to an association between IMCL and decreased Akt phosphorylation (p = 0.059), despite no association with insulin resistance. In turn, hepatic steatosis (p = 0.015) and inflammation (p = 0.013) were associated with decreased Akt phosphoryation. Citrate synthase activity was lower in obese patients (p = 0.047) whereas complex I (p = 0.040) and III (p = 0.036) activities were higher, compared with controls. Finally, in obese patients, complex I activity increased with progressive steatosis (p = 0.049) and with a trend with fibrosis severity (p = 0.056).

**Conclusions:**

In morbid obese patients, presence of IMCL associates with NASH and advanced fibrosis. Muscle mitochondrial dysfunction does not appear to be a major driving force contributing to muscle fat accumulation, insulin resistance or liver disease. Importantly, insulin resistance in muscle might occur at a late point in the insulin signaling cascade and be associated with IMCL and NAFLD severity.

## Introduction

Nonalcoholic fatty liver disease (NAFLD) is a condition characterized by fat accumulation in the liver, not related with alcohol consumption. It represents a wide spectrum of pathological subgroups, from benign simple steatosis to nonalcoholic steatohepatitis (NASH), which can progress to hepatic cirrhosis [1], and is associated with overall and liver-related increased mortality [2]. Two of the main risk factors for developing NAFLD are insulin resistance and obesity, in which the peripheral adipose tissue reservoir capacity is overwhelmed, allowing ectopic fat accumulation.

There is increasing awareness of the importance of the muscle as a central player in the adaptation to an excessive input of energy, as one of the main fuel consuming organs [3]. In the muscle, lipids are stored either as metabolically inert interstitial adipocyte triglycerides in the interfascicular space, extramyocellular lipids (EMCL), or droplets in the cytoplasm of myocytes, intramyocellular lipids (IMCL) [4]. IMCL accumulate in athletes, where they are in constant turnover and believed to act as fuel [5], or in association with obesity, insulin resistance/type 2 diabetes mellitus and high fat diets [6], [7], [8], [9]. In this case, lipids are either not consumed or incompletely oxidized, potentially forming dangerous active metabolites that may inhibit the insulin signaling cascade [10], [11], [12], [13], [14], thus further contributing to metabolic disturbances. In fact, IMCL may constitute the missing link between obesity and development of insulin resistance, since overweight individuals can improve their insulin sensitivity with exercise training, even in the absence of significant changes in total body adiposity [15]. In addition, interventions in obese individuals that decrease IMCL result in better glucose control [16]. Nonetheless, the accumulation of active fatty acids metabolites such as acyl CoA, ceramides and diacylglycerol, rather than the accumulation of triglycerides, may play a major role in insulin signaling impairment. In fact, the up-regulation of triglyceride synthesis was able to protect skeletal muscle from fat-induced insulin resistance in a mouse model [17].

Previous studies with indirect assessment of ectopic fat in the liver and muscle, either by computer tomography scan [18] or magnetic resonance spectroscopy [7], have suggested that fat accumulation in these two tissues may be correlated. Therefore, we aimed to evaluate skeletal muscle changes in morphology, mitochondrial function and insulin signaling, in morbidly obese patients, and correlate them with NAFLD severity.

## Methods

### Patients

A cross-sectional study was performed with prospective and consecutive recruitment of morbid obese patients submitted to bariatric surgery, at Hospital of Santa Maria, CHLN, Lisbon, Portugal, from 2006 to 2009. Inclusion criteria were age higher or equal to 18 years old and indication to bariatric surgery. This was defined as body mass index (BMI) superior or equal to 40 kg/m^2^ or superior to 35 kg/m^2^ if associated with significant morbidity related with obesity [19], such as arterial hypertension, diabetes mellitus type 2, obstructive sleep apnea or dyslipidemia. Exclusion criteria were: significant alcohol consumption defined as superior to 20 grams per day; positivity to hepatitis B virus surface antigen; positivity to anti-hepatitis C virus; other type of liver diseases namely primary biliary cirrhosis, autoimmune hepatitis, primary sclerosing cholangitis, Wilson's disease, hemochromatosis or α1-antitripsin deficiency; treatment with potentially steatogenic drugs such as steroids, high-dose estrogen, tamoxifen, methotrexate or amiodarone within six months of enrollment and history of gastrointestinal bypass surgery or segmental small bowel resection. The study protocol conformed to the Ethical Guidelines of the 1975 Declaration of Helsinki, revised in 2000, as reflected in an a priori approval by the Hospital de Santa Maria Human Ethics Committee and written informed consent was obtained from all participants.

### Clinical assessment and laboratorial tests

Patients were submitted to an interview assessing past medical history and a semi-structured questionnaire regarding alcohol consumption. BMI was defined as an individual's weight in kilograms divided by the square of height in meters (kg/m^2^). Waist was measured at half way between the inferior rib and the iliac crest and hip was measured at the gluteus muscle at the level of maximum circumference. Hypertension was defined as a systolic blood pressure greater than or equal to 140 mmHg, a diastolic blood pressure greater than or equal to 90 mmHg or as being treated with antihypertensive drugs [20].

Venous blood was drawn in the morning after an overnight fast. Laboratorial assessment included liver biochemistry, plasma concentrations of cholesterol, high-density lipoprotein-cholesterol (HDL-C), triglycerides, insulin and glucose. LDL-cholesterol was calculated by the Friedewald's equation i.e. [total cholesterol – HDL-cholesterol – (triglycerides/5)]. Homeostasis model assessment of insulin resistance (HOMA-IR) was calculated using fasting glucose and insulin measurements: (fasting insulin (mU/mL)×fasting glucose (mmol/L)/22.5) [21]. Insulin resistance was considered if HOMA was greater than or equal to 3 [22]. Diabetes mellitus was defined as fasting blood glucose greater than or equal to 126 mg/dL (7 mmol/L), or by the regular use of hypoglycemic medications [23]. Metabolic syndrome was defined according to the Third Adult Treatment Panel of the National Cholesterol Education Program, as the presence of at least three of the following criteria: central obesity (waist circumference greater than 102 cm in men and 88 cm in women), hypertriglyceridemia (greater than or equal to 150 mg/dL), low HDL-cholesterol (lower than 40 mg/dL in men and 50 mg/dL in women), elevated fasting glucose (higher than 110 mg/dL) or the use of hipoglicemic drugs and high blood pressure (higher or equal to 130/85 mmHg or under antihypertensive drugs) [24].

Serum levels of adiponectin, leptin and ghrelin were assessed by enzyme-linked immunosorbent assay (ELISA) or radioimmunoassay (RIA). In order to minimize individual variations, serum levels were determined in duplicate.

### Liver Biopsies

At the time of surgery, all patients were submitted to wedge liver biopsy. All biopsies were evaluated by the same experienced hepatopathologist, blinded to the laboratorial parameters and clinical data. The specimens were fixed in formalin, embedded in paraffin and stained with hematoxylin eosin, Masson trichrome and reticulin coloration. NAFLD was defined as the presence of more than 5% of steatosis in the liver [25] and NASH was defined as any degree of steatosis along with centrilobular ballooning and/or Mallory-Denk bodies or any degree of steatosis along with centrilobular pericellular/perisinusoidal fibrosis or bridging fibrosis in the absence of another identifiable cause [26]. Steatosis severity was graded from 0 to 3 according to steatosis in less than 5% of hepatocytes, 5–33%, 33–66% and more than 66%; hepatocyte ballooning was classified from 0 to 2, namely absent, few or many; lobular inflammation was classified from 0 to 3, according to the presence of no inflammatory foci, mild or less than 2 foci per 200× field, moderate or 2–4 foci per 200× field and severe or more than 4 foci per 200× foci. Finally, fibrosis was staged from 0 to 4, according to no fibrosis, perisinuoidal or periportal, perisinusoidal and periportal, bridging fibrosis or cirrhosis, as described by the Pathology Committee of NASH Clinical Research Network [27].

### Deltoid Muscle Biopsies

Deltoid muscle samples from 51 morbidly obese patients with NAFLD and 34 lean healthy controls were evaluated. Left deltoid muscle biopsies were obtained during surgery, with collection of three fragments, of about 10×5 mm in size, which were immediately flash frozen in 2-methylbutan cooled in liquid nitrogen and kept at −80°C. Two fragments were used for histological evaluation and biochemistry.

Histological evaluation of muscle biopsies included myocyte fibers morphology, lipid and glycogen content, inflammation and necrosis, as well as mitochondrial abnormalities. Morphological characterization of muscle fibers was performed using the ATPase technique, with pre-incubation at pH 9.4 that allows differentiation according to the intensity of the stain; light fibers were classified as type I and dark fibers as type II. To evaluate the proportion of each type of fiber, computerized counting of 400 consecutive muscle fibers was performed using informatics program Motic Images Advanced 3.1. In addition, the lower diameter of 150 consecutive muscle fibers was measured to evaluate length variation. Semi-quantitative assessment of lipid content was performed with the Oil Red stain, to differentiate intramyocellular and/or extramyocellular and interfascicular lipids overload. Glycogen content was assessed with the Periodic-Acid-Schiff (PAS) stain. The assessment of mitochondrial abnormalities included the determination of ragged red fibers (RRF) with modified Gomori Trichrome stain, which allows the identification of a peripheral reddish discoloration translating excessive mitochondrial proliferation; and cytochrome c oxidase (COX) negative fibers, through histoenzymatic evaluation with the identification of fibers with no COX activity as a result of mitochondrial DNA mutations that predominantly compromise complex IV of the mitochondrial respiratory chain.

The mitochondrial respiratory chain enzymes activities were evaluated with an enzymatic assay through spectrophotometric analysis to determine complex I (NADH-ubiquinone oxidoreductase), II (succinate-ubiquinone reductase), III (ubiquinol-cytochrome c reductase) and IV (cytochrome c oxidase), as well as citrate synthase, activities. Each complex enzymatic assay was normalizated with the citrate synthase activity of each sample. Assays were performed using cellular homogenates of 50–80 mg of muscle. Three assays for each enzyme in each sample were done using a spectrophotometer Shimadzu CPS-240A and the informatics program PC160PLS.

### Muscle Insulin Signaling Cascade

Muscle samples from 26 patients were used to evaluate insulin signaling cascade through immunoblotting. Total protein extracts were subjected to SDS-PAGE electrophoresis [28]. Blots were incubated with primary rabbit polyclonal antibodies against insulin receptor, phosphorylated insulin receptor Tyr1162/1163, insulin receptor substrate-1 (IRS-1), phosphorylated IRS-1 Tyr632, or with primary mouse monoclonal antibodies reactive to Akt and phosphorylated Akt Ser473 (Santa Cruz Biotechnology, Santa Cruz, CA, USA), and finally with secondary antibodies conjugated with horseradish peroxidase (Bio-Rad Laboratories, Hercules, CA, USA). Levels of phosphorylated proteins were normalized to corresponding unphosphorylated proteins. Glyceraldehyde-3-phosphate dehydrogenase was used as loading control. Membranes were processed for protein detection using Super Signal substrate (Pierce, Rockford, IL, USA). The relative intensities of protein bands were analyzed using a densitometry analysis program (Quantity One Version 4.6; Bio-Rad Laboratories).

### Statistical Analysis

Basic descriptive statistics means, standard deviation, ranges and percentages, were used to characterize the different populations. Qualitative variables were analyzed by χ^2^ test and one-way ANOVA. Results are also presented as odds ratio (OR) with 95% confidence interval (CI). Quantitative variables were compared by unpaired Student's t-test and one-way ANOVA and correlations by Pearson correlation coefficient. The independence of the associations of variables with the presence of NASH (dependable variable) was assessed by multivariable logistic regression analysis and expressed as odds ratio (OR). The covariates included were selected by their statistical association in univariate analysis. Analyzes were run using SPSS software version 16.0 (SPSS, Chicago, IL) for Windows and GraphPad InStat version 3.00 (GraphPad Software, San Diego, CA, USA) for the analysis of variance and Bonferroni's multiple comparison tests. Two-tailed P values less than 0.05 were considered statistically significant.

## Results

### Patients' characteristics

**Figure 1:**
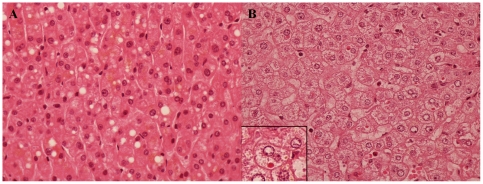
Liver histology. Slides stained with hematoxylin & eosin representing patients with simple steatosis (20×) (A) and NASH (40×) (B). Note the presence of a ballooned hepatocyte and lobular inflammatory infiltrate. Insert: ballooned hepatocyte with a Mallory-Denk body and megamitochondria.

Fifty one morbid obese patients with NAFLD were included, of whom 22 (43%) were classified as NASH (Typical case - Figure 1 B) and 29 (57%) as non-NASH (Typical case - Figure 1 A). One patient (4%) had unsuspected hepatic cirrhosis. Thirteen patients were male and mean body mass index was 45±6 kg/m^2^. Insulin resistance was present in 57%, diabetes mellitus 26%, arterial hypertension 39% and metabolic syndrome 43%. Main characteristics of the patient population are showed in Table 1.

**Table 1:** Patients main features.

Characteristic	Total (N = 51)	NASH (N = 22)	No NASH (N = 29)	P*	OR [95%CI]
Age (years)	42±10	45±9	41±11	0.105	
Male sex (%)	26	32	21	0.280	0.56 [0.16–2.00]
BMI (kg/m^2^)	45±6	45±6	46±7	0.739	
Waist (cm)	132±13	133±17	134±17	0.930	
WTH ratio	0.97±0.10	1.01±0.10	0.96±0.11	0.095	
Diabetes mellitus (%)	26	27	24	0.525	1.18 [0.33–4.18]
Insulin resistance (%)	57	79	44	**0.020**	**4.77** [1.23–18.53]
HOMA	3.9±2.3	4.7±2.4	3.3±2.0	**0.049**	
Hypertension (%)	39	41	38	0.528	1.13 [0.36–3.52]
Smoking (%)	30	24	34	0.311	0.59 [0.17–2.10]
Sleep apnea (%)	23	23	24	0.588	0.92 [0.25–3.43]
Hypertrigliceridaemia (%)	19	25	15	0.305	1.92 [0.44–8.41]
Triglycerides (mg/dL)	119±43	127±49	112±37	0.223	
HDL-cholesterol (mg/dL)	51±13	48±12	53±13	0.146	
LDL-cholesterol (mg/dL)	122±36	128±30	117±40	0.293	
Metabolic syndrome (%)	43	59	30	**0.037**	**3.43** [1.15–11.22]
AST (IU/L)	27±20	31±29	24±9	0.272	
ALT (IU/L)	33±29	41±42	27±12	0.141	
γ-GT (IU/L)	37±45	54±64	25±14	**0.022**	
ALP (IU/L)	74±22	73±26	71±14	0.306	
Total bilirubin (mg/dL)	0.7±0.4	0.6±0.2	0.7±0.4	0.274	
Adiponectin (ng/mL)	20.7±6.2	21.1±7.7	20.4±5.3	0.798	
Leptin (ng/mL)	19.8±7.3	19.9±7.3	19.7±7.5	0.912	
Ghrelin (pg/mL)	20.8±13.1	18.8±13.9	21.9±12.8	0.481	
NAS score	2.7±1.6	3.2±1.8	2.4±1.3	0.105	
Steatosis grade	1.8±0.8	1.9±0.8	1.7±0.7	0.309	
1 (n)	21	8	13		
2 (n)	18	7	11		
3 (n)	13	7	5		
Hepatocelular ballooning (n)	3	2	1	0.379	
Lobular inflammatory grade	0.8±0.8	1.1±0.9	0.6±0.8	0.084	
0 (n)	23	7	16		
1 (n)	16	8	8		
2 (n)	11	6	5		
3 (n)	1	1	0		
Fibrosis stage	1.4±0.8	2.1±0.5	0.9±0.3	<0.001	
0 (n)	4	0	4		
1 (n)	25	0	25		
2 (n)	20	20	0		
3 (n)	1	1	0		
4 (n)	1	1	0		

NASH = non alcoholic steatohepatitis, BMI = body mass index, WTH = waist to hip, AST = aspartate aminotransferase, ALT = alanine aminotransferase, γ-GT = γ-glutamyl transpeptidase, ALP = alkaline phosphatase, NAS = NAFLD activity score.

P* = significance level in the comparison between NASH and non NASH.

### Metabolic factors and hepatic histology

Age, male∶female ratio and anthropometric parameters including BMI were similar in NASH and non-NASH patients. NASH was associated with the presence of insulin resistance (OR 4.76 [1.23–18.52], p = 0.045) and metabolic syndrome (OR 3.41 [1.15–11.22], p = 0.038), as well as higher levels of gamma-glutamyl transpeptidase (γ-GT) (Table 1). In multivariate analysis, the metabolic syndrome lost statistical significance. Plasma levels of adipokines (adiponectin, leptin) and ghrelin were not associated with NASH.

**Figure 2:**
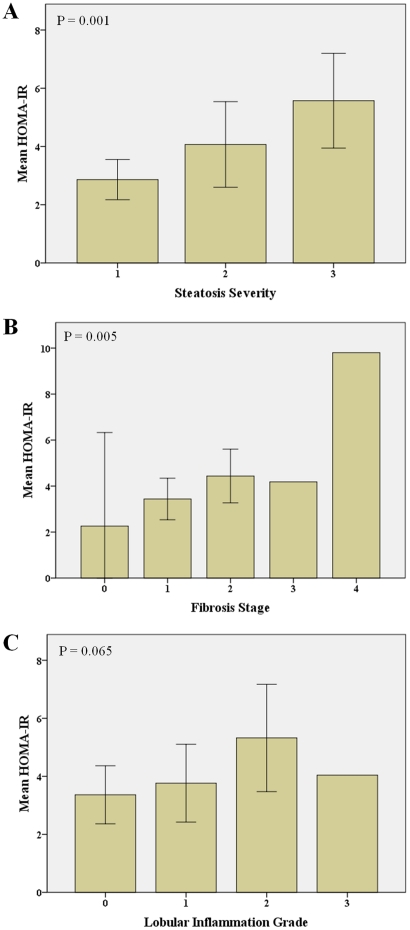
Insulin resistance according to liver histology. Differences in HOMA-IR according to the severity of steatosis (A), fibrosis stage (B) and lobular inflammatory grade (C). Error bars: 95% CI (not possible to calculate for fibrosis stage 3 or 4 or lobular inflammation grade 3, only one patient). HOMA-IR progressively increased with severity of hepatic steatosis, lobular inflammation and fibrosis.

Steatosis severity paralleled a progressive increase in insulin levels (p = 0.002), mean HOMA-IR (p = 0.001) and prevalence of insulin resistance (p = 0.009) (Figure 2 A). Also, steatosis severity was associated with progressively higher levels of aminotransferases serum levels, aspartate – AST (p = 0.011) and alanine aminotransferase – ALT (p = 0.001). Similarly, there was a statistical positive association between fibrosis stage and higher levels of γ-GT (p = 0.003), insulin (p = 0.006) (Figure 2) and HOMA-IR (p = 0.005) (Figure 2B), as well as progressively higher prevalence of insulin resistance (p = 0.009) and the metabolic syndrome (p = 0.029). There was also a trend to a positive association between age and fibrosis severity (p = 0.054). Lastly, progressive lobular inflammatory activity was not associated with HOMA-IR (p = 0.065) (Figure 2C) or insulin resistance (p = 0.120), but was associated with older age (p = 0.001), AST (p<0.001) and ALT serum levels (p<0.001), as well as higher prevalence of the metabolic syndrome (p = 0.031).

### Muscle morphology in morbidly obese NAFLD patients and normal lean controls

Deltoid muscle morphology was evaluated in 34 lean healthy controls and 51 morbidly obese patients with NAFLD.

There were no differences in number, proportion, length and length variability of type 1 and 2 fibers in controls and NAFLD patients, as well as NASH and non-NASH obese patients. However, severity of hepatic steatosis was associated with progressively higher type 1 (p = 0.021) and type 2 (p = 0.007) fiber length. No associations with fibrosis stage or lobular inflammatory grade were found.

**Figure 3:**
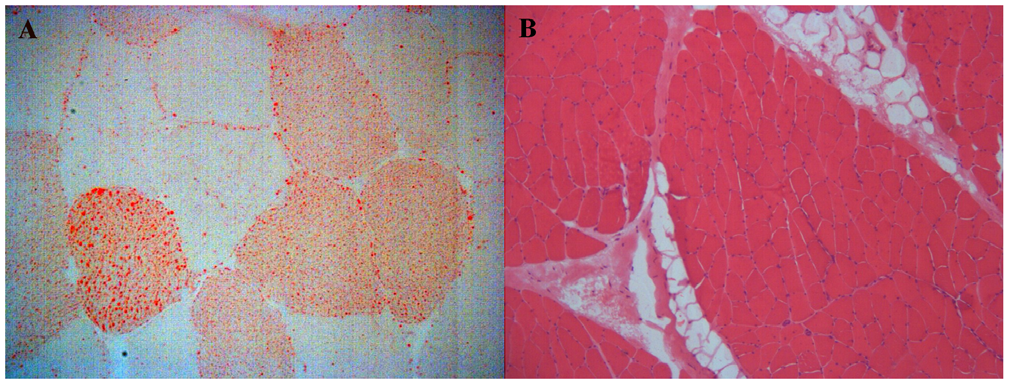
Deltoid muscle histology. Slides showing intramyocellular fat accumulation highlighted in Oil red, (40×) (A) and inter fascicular fat, stained with hematoxylin & eosin (10×) (B).

**Figure 4:**
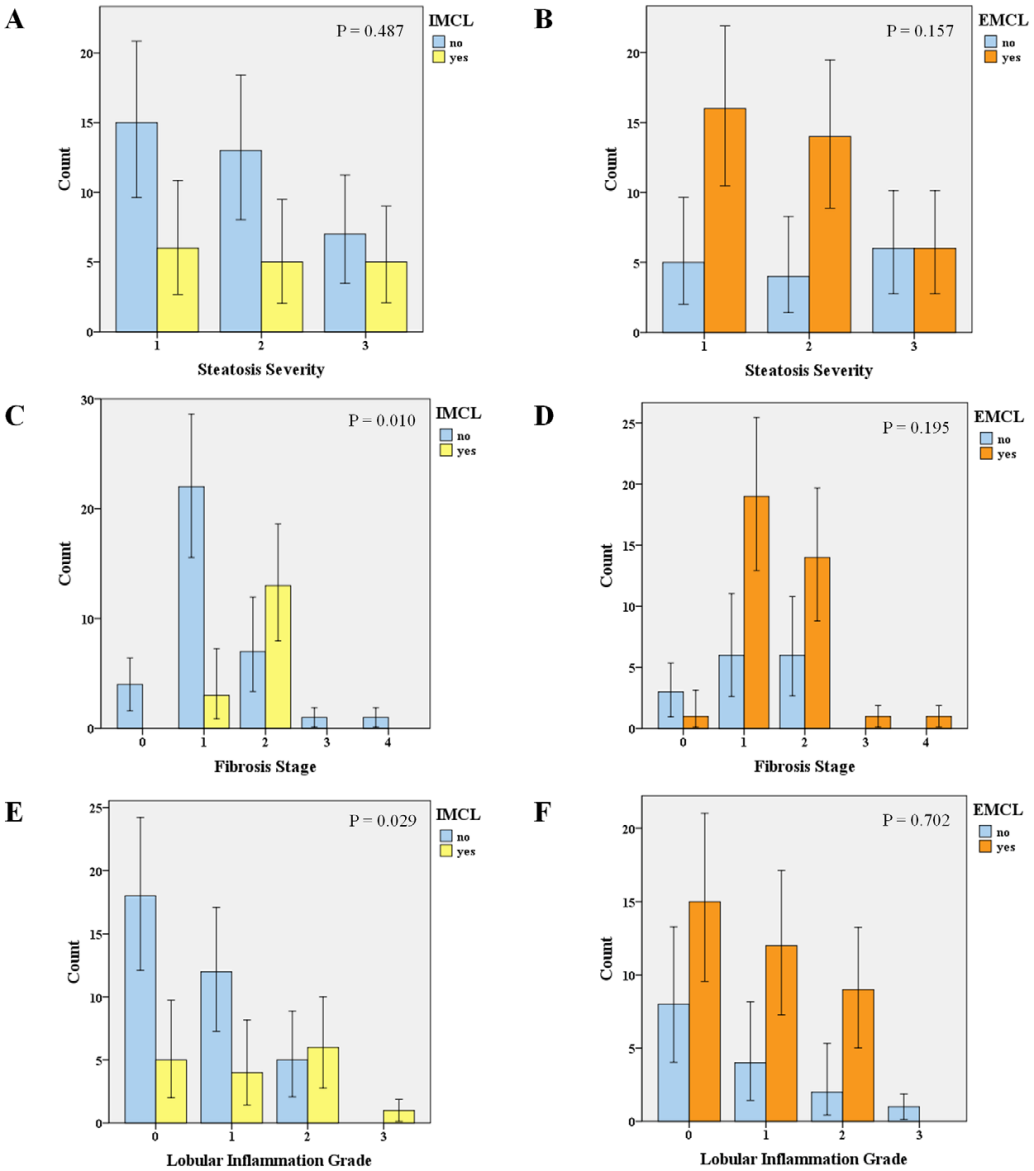
Distribution of muscle fat according to liver histology. Prevalence of IMCL and EMCL according to steatosis severity (A and B), fibrosis stage (C and D) and lobular inflammation grade (E and F). Error bars: 95% CI. IMCL was progressively more prevalent with more severe liver disease, namely fibrosis and lobular inflammation. EMCL did not associate with hepatic histology.

The presence of EMCL and IMCL (Figure 3B and 3A respectively) was more frequent in obese NAFLD patients as compared to controls (70.6% *vs* 8.8%, p<0.001 and 31.4% *vs* 0%, p<0.001, respectively). Also, although the percentage of patients presenting EMCL was not different between NASH and non-NASH obese patients, the percentage of patients with IMCL was significantly higher in NASH patients (59.1 *vs* 10.3, p<0.001; OR 12.52 [2.89–54.26], p<0.001). Furthermore, even though there was no significant association between IMCL and steatosis severity, there was a significant tendency to a progressive increase in the prevalence of IMCL and the severity of fibrosis stage (p = 0.010) and lobular inflammation grade (p = 0.029). In turn, no association was found between EMCL and liver histology (Figure 4). Surprisingly, IMCL or EMCL were not associated with metabolic factors, namely BMI, waist to hip ratio, insulin resistance or HOMA-IR, hypertension, diabetes mellitus, dyslipidemia or the metabolic syndrome. Furthermore, IMCL or EMCL did not change in patients under treatment with anti-diabetics or statins, nor with cytokines levels, namely adiponectin, leptin or ghrelin.

In morbidly obese patients, as well as in controls, there was no significant muscle inflammation or necrosis, irrespective of the presence of IMCL. Lean controls presented more mitochondrial aggregates, and less cytochrome c oxidase negative myocytes and glycogen overload. However, no significant differences were observed between NASH and non-NASH obese patients (Table 2). Still, there was a tendency, though with no statistical significance, to a higher percentage of glycogen overload prevalence with increasing steatosis (29% *vs* 39% *vs* 59%, p = 0.101). Also, the prevalence of cytochrome c oxidase negative fibers increased gradually with lobular inflammation activity severity (p = 0.045). No other associations, namely with steatosis, fibrosis or lobular inflammation severity were found.

**Table 2:** Muscle morphology in morbidly obese patients with NAFLD and lean controls.

Feature	Controls (N = 35)	Obese (N = 51)	P*	NASH (N = 22)	Non-NASH (N = 29)	P**
T1F (N)	200±37	215±38	0.102	215±37	214±40	0.894
T1F proportion (%)	50±9	54±10	0.102	54±9	53±10	0.894
T1F length (µm)	52.5±8.3	55.6±10.0	0.154	55.1±8.6	55.9±11.1	0.792
T1F Δ coefficient	172.1±27.7	182.4±20.8	0.069	185.7±20.9	179.9±20.8	0.378
T2F (N)	202±44	185±38	0.076	184±37	186±40	0.894
T2F proportion (%)	50±11	46±10	0.076	46±9	47±10	0.894
T2F length (µm)	52.5±10.2	52.8±11.6	0.911	52.0±8.5	53.3±13.7	0.708
T2F Δ coefficient	219.9±103.1	215.3±31.0	0.783	217.4±35.1	213.6±28.3	0.698
EMCL (%)	8.8	70.6	**<0.001**	72.7	69.0	0.510
IMCL (%)	0	31.4	**<0.001**	59.1	10.3	**<0.001**
RRF (%)	8.8	21.6	0.103	18.2	24.1	0.437
Mitochondrial aggregates	79.4	51	**0.007**	45.5	55.5	0.689
COX (%)	5.9	39.2	**<0.001**	45.5	34.5	0.306
Inflammation	0	2.0	0.600	0	3.4	0.569
Necrosis	2.9	7.8	0.331	9.1	6.9	0.814
Glycogen overload	0	39.2	**<0.001**	40.9	37.9	0.528

NASH = non alcoholic steatohepatitis, T1F = type 1 fibers, Δ coefficient = variability coefficient, T2F = type 2 fibers, EMCL = extramyocellular lipids, EMCL = intramyocellular lipids, RRF = ragged red fibers presence, COX = presence of cytochrome oxidase negative fibers.

P* = significance level in the comparison between controls and obese patients;

P** = significance level in the comparison between NASH and non NASH.

### Muscle mitochondrial enzymes activity in morbidly obese NAFLD patients and normal lean controls

Muscle mitochondrial activity of citrate synthase and respiratory chain complex I, II, III and IV were evaluated in 25 lean healthy controls and 42 morbidly obese patients with NAFLD.

In obese patients, there was a lower citrate synthase activity compared with lean controls, translating into a decreased mitochondrial content (101±30 *vs* 117±34 nmol/min/mg/PNC, p = 0.047). Moreover, complex I and III activities were higher in obese NAFLD patients compared with controls (20±10 *vs* 16±5 nmol/min/mg/PNC and 87±46 *vs* 68±36 nmol/min/mg/PNC, p<0.05 respectively). There were no differences in enzymatic activities between obese groups (Table 3).

**Table 3:** Muscle mitochondrial respiratory chain enzymes activity in morbidly obese patients with NAFLD and lean controls.

Feature	Controls (N = 25)	Obese (N = 42)	P*	NASH (N = 18)	Non-NASH (N = 24)	P**
Citrate Synthase	117.3±34.0	101.3±29.7	**0.047**	109.2±32.7	95.3±26.5	0.138
Complex I	16.2±5.3	20.3±10.5	**0.040**	22.6±12.1	18.6±9.0	0.223
Complex II	17.0±3.8	15.6±6.3	0.242	16.6±5.4	14.8±6.9	0.364
Complex III	67.7±35.7	87.4±46.3	**0.036**	83.0±43.7	90.6±48.8	0.604
Complex IV	18.2±5.3	17.7±7.7	0.755	17.2±7.0	18.0±8.4	0.738

NASH = non alcoholic steatohepatitis. All enzymatic activities expressed in nmol/min/mg PNC.

P* = significance level in the comparison between controls and obese patients;

P** = significance level in the comparison between NASH and non NASH.

There were also no differences in enzymatic activities regarding the presence of IMCL. On the contrary, the presence of EMCL was globally associated with lower citrate synthase (99±16 *vs* 115±32 nmol/min/mg/PNC, p = 0.047) and complex II (15±6 *vs* 18±5 nmol/min/mg/PNC, p = 0.026) activities, and higher complex III activities (91±50 *vs* 69±31 nmol/min/mg/PNC, p = 0.043). However, differences were lost when analyzing obese patients with NAFLD alone.

In addition, in patients with insulin resistance, complex I activity was higher (24±11 *vs* 16±18 nmol/min/mg/PNC, p = 0.023) whereas complex IV (15±4 *vs* 21±9 nmol/min/mg/PNC, p = 0.036) was lower. Also, complex I activity positively correlated with glucose (r = 0.38, p = 0.029) and insulin (r = 0.40, p = 0.018) plasma levels, although there were no differences regarding the presence of diabetes mellitus or metabolic syndrome. Surprisingly, adiponectin and complex III activity presented a negative correlation (r = −0.41, p = 0.014).

**Figure 5:**
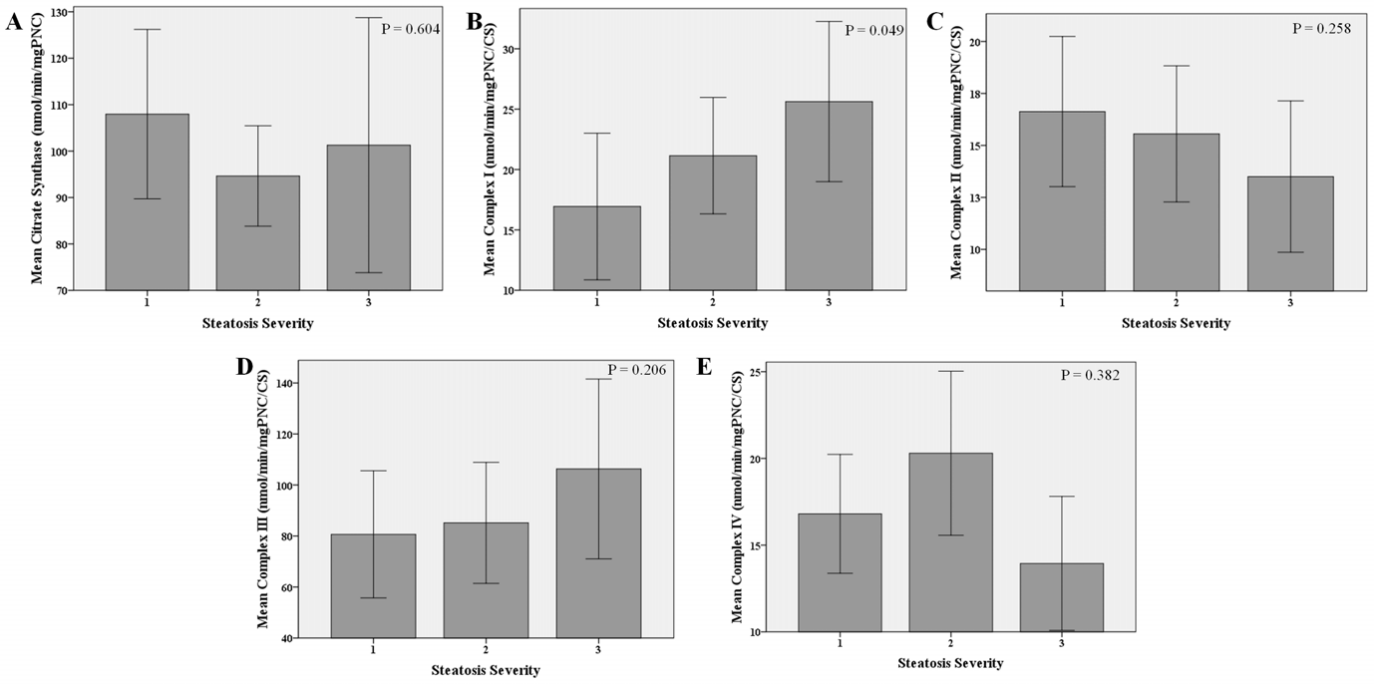
Muscle mitochondrial enzymatic activities according to hepatic steatosis degree. Differences in mean mitochondrial enzymatic activities, citrate synthase (A), complex I (B), complex II (C), complex III (D) and complex IV (E) with steatosis severity. Error bars: 95% CI. Complex I activity progressively increased with hepatic steatosis severity.

**Figure 6:**
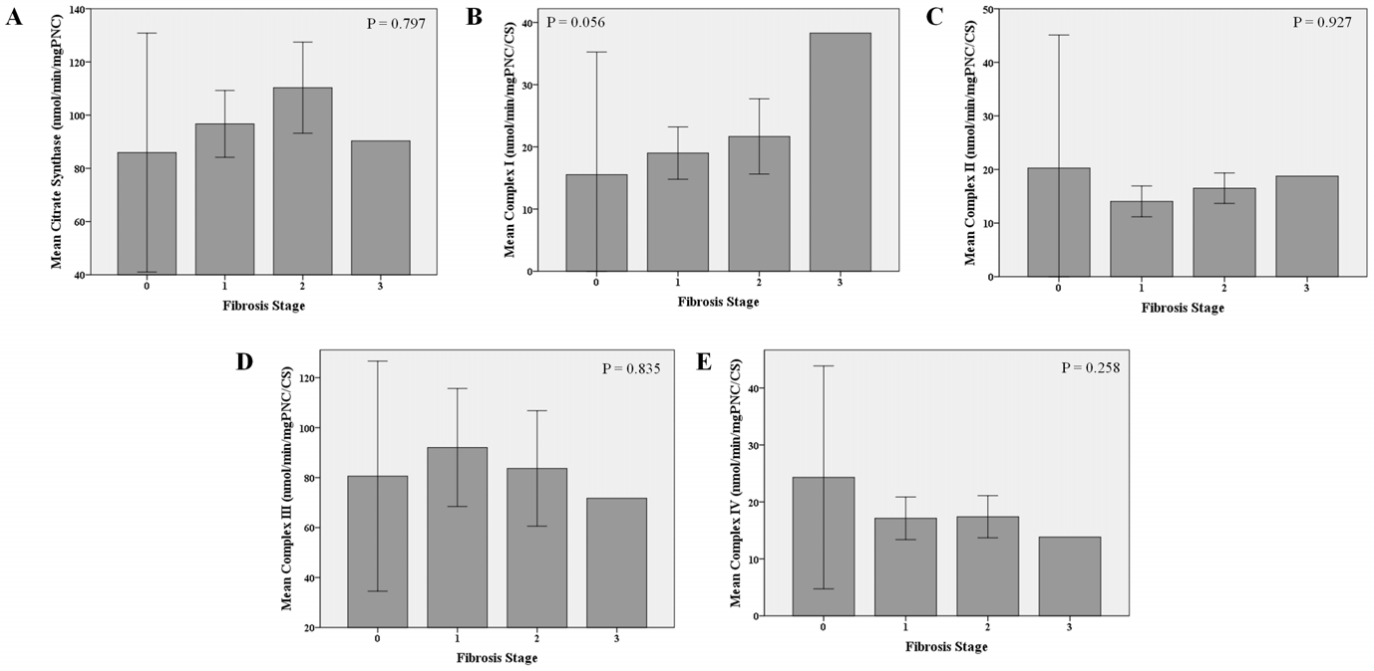
Muscle mitochondrial enzymatic activities according to hepatic fibrosis severity. Differences in mean mitochondrial enzymatic activities, citrate synthase (A), complex I (B), complex II (C), complex III (D) and complex IV (E) with fibrosis severity. Error bars: 95% CI. There was a trend to a progressive increase in complex I activity according to fibrosis stage.

**Figure 7:**
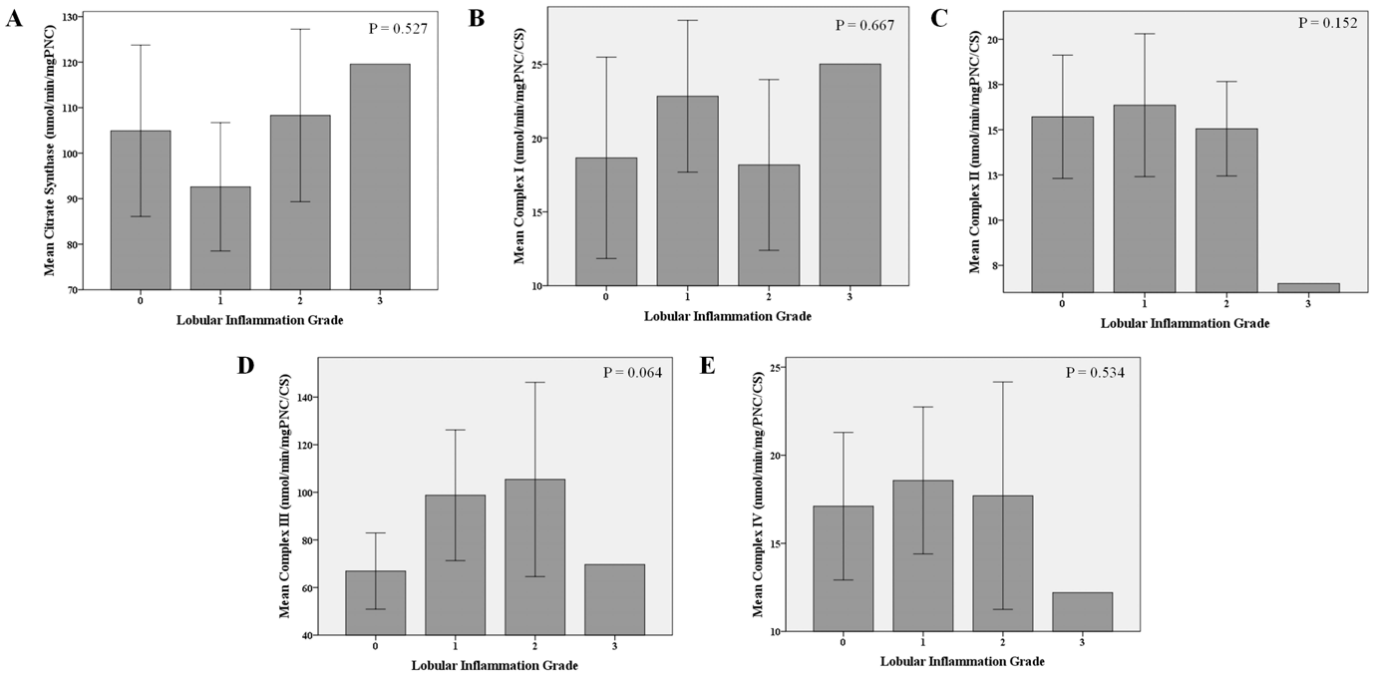
Muscle mitochondrial enzymatic activities according to hepatic inflammation severity. Differences in mean mitochondrial enzymatic activities, citrate synthase (A), complex I (B), complex II (C), complex III (D) and complex IV (E) with lobular inflammation severity. Error bars: 95% CI. There was a trend to a progressive increase in complex III activity according to lobular inflammation grade.

Concerning liver lesion, complex III activity was positively correlated with ALT levels (r = 0.35, p = 0.024) whereas complex IV negatively correlated with AST (r = −0.41, p = 0.008) and ALT (r = −0.33, p = 0.031) levels. Complex I activity gradually increased with progressive steatosis (p = 0.049) and fibrosis severity (p = 0.056). No other associations were found between enzymatic activities and steatosis, fibrosis or lobular inflammation severity (Figures 5, 6 and 7).

### Muscle insulin signaling cascade in morbidly obese NAFLD patients and its relation with muscle and hepatic morphology

The insulin signaling cascade in the muscle was evaluated in 26 morbid obese patients with NAFLD, of whom 43% had NASH, 59% insulin resistance and 26% type 2 diabetes mellitus. No differences were found between insulin cascade proteins and the presence of insulin resistance assessed by HOMA-IR, diabetes mellitus or the metabolic syndrome. However, there was a trend to a positive correlation between glucose plasma levels and phosphorylated IRS-1 (r = 0.480, p = 0.0913) and a negative correlation with HDL-cholesterol plasma levels (r = −0.393, p = 0.057). Regarding BMI, there was a positive correlation with increased phosphorylated IRS-1 (r = 0.388, p = 0.043) and a trend towards a negative correlation with total Akt levels (r = −0.336, p = 0.093). Surprisingly, no correlations were found with adiponectin. However, phosphorylated IRS-1 was positively correlated with leptin (r = 0.393, p = 0.047), while the insulin receptor protein levels were positively correlated with ghrelin levels (r = 0.447, p = 0.042).

Although there were no differences regarding the presence of EMCL and the insulin cascade, IMCL was associated with a 1.5 fold decrease in phosphorylated Akt levels (p = 0.059). The only correlations found with respiratory chain enzymes activities were a positive correlation between complex I and phosphorylated insulin receptor (r = 0.537, p = 0.007) and a trend to a negative correlation between complex III and phosphorylated Akt levels (r = −0.378, p = 0.069).

**Figure 8:**
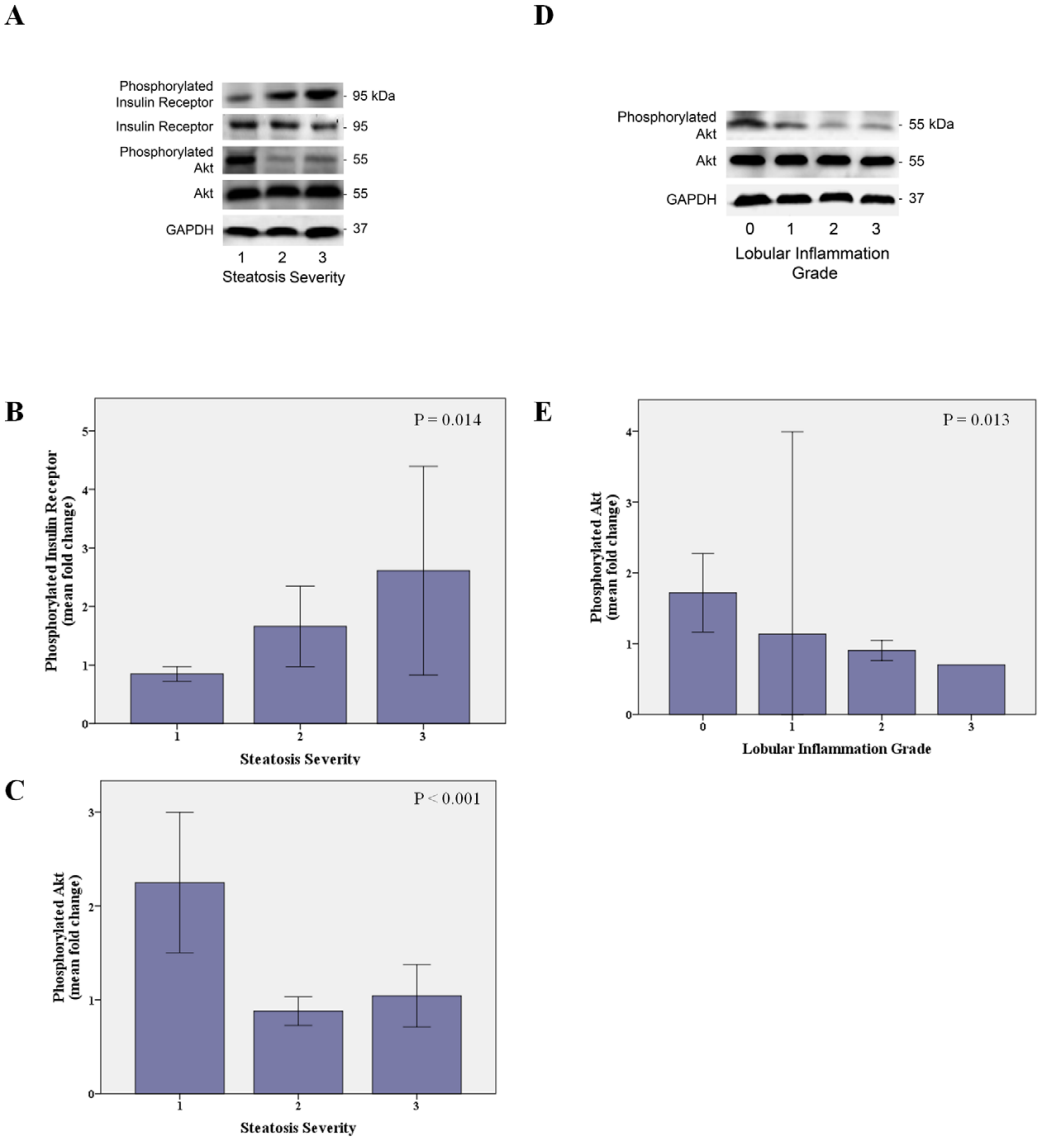
Muscle insulin receptor pathway according to hepatic histology. Changes in phosphorylated insulin receptor and Akt in muscle tissue with hepatic steatosis severity (A). Insulin receptor phosphorylation in the muscle increases with hepatic steatosis severity (B) and Akt phosphorylation decreases with steatosis (C). Changes in phosphorylated Akt in muscle tissue with hepatic lobular inflammation grade (D). Akt phosphorylation in the muscle decreases with lobular inflammation grade (E). Total proteins were extracted for immunoblot analysis as described. Representative immunoblots for insulin receptor, phosphorylated insulin recptor, Akt, and phosphorylated Akt are shown. Tissue blots of phosphorylated proteins were normalized to corresponding unphosphorylated proteins. Glyceraldehyde-3-phosphate dehydrogenase (GAPDH) was used as loading control. Error bars: 95% CI.

Concerning hepatic histology, patients with at least moderate steatosis presented a 2.4-fold increase in phosphorylated insulin receptor (p = 0.005), a trend to a 1.4-fold increase in phosphorylated IRS-1 (p = 0.052), a 2.7-fold increase in total Akt (p = 0.015), and a 2.4-fold decrease in phosphorylated Akt levels (p = 0.004). In addition, there was a gradual increase of phosphorylated insulin receptor (p = 0.014) and a gradual decrease of phosphorylated Akt (p<0.001), with increased steatosis severity (Figure 8). Similarly, the presence of at least moderate lobular inflammation associated with a 2.1-fold decrease in total Akt (p = 0.043) and a 1.9-fold decrease in phosphorylated Akt (p = 0.005). Finally, lobular inflammation severity was associated with progressive decrease in phosphorylated Akt (p = 0.013), while the presence of fibrosis was only associated with a 2-fold increase in phosphorylated insulin receptor levels (p = 0.008).

## Discussion

To our knowledge, this is the first study that evaluates the interplay between muscle and hepatic histology in morbid obese patients. As expected, morbid obese patients presented a higher prevalence of fatty muscle than lean controls. However, unlike the liver, in which ectopic fat may be associated with necroinflammation and fibrosis progression our results suggest that ectopic fat in the muscle does not seem to exhibit a predominant toxic role. In fact, no evidence of muscle inflammation or cellular necrosis was found in histology assessment. Previous studies had shown an association between IMCL and hepatic steatosis [7], [18]. In the present study, we further demonstrated that IMCL associates with a more than 12-fold risk for NASH in morbid obese patients, as well as with hepatic inflammation and advanced fibrosis. Indeed, IMCL may translate into a higher susceptibility to lipotoxicity and a decreased ability to keep lipids in inert reservoirs. It would be interesting to understand if there is a temporal sequence between IMCL and NASH development with one favoring the other or if both are parallel phenomena that share the same risk factors. For that, sequential biopsies would be necessary, and hence only possible to study in animal models. Although IMCL was not associated with increased HOMA-IR or overt diabetes mellitus, it was associated with impairment in muscle insulin signaling cascade. Therefore, IMCL may constitute a more sensitive and earlier marker of glucose metabolism disturbance. Finally, IMCL did not associate with muscle mitochondrial dysfunction, thus suggesting ectopic fat accumulation to be a result of an excess of fatty acids supply rather than an impairment in mitochondrial fatty acids oxidation [29].

Our findings also show a decrease in muscle mitochondrial content, as assessed by citrate synthase activity in obese patients as compared to controls. However, we did not find any signs of mitochondrial dysfunction. On the opposite, obese patients presented increased complex I and III activities. Previous studies had shown that mitochondrial content and fatty acids oxidation are decreased in obesity [30], [31]. This may be explained by lipid-derived oxidative mitochondrial damage [32], [33]. Alternatively, mitochondrial content may decrease as a result of sedentary lifestyle rather than obesity itself [34], [35], [36], [37]. However, it has also been previously described that isolated mitochondria from obese subjects maintain its ability to oxidize fatty acids [32]. There may be, indeed, a compensatory increase in mitochondrial enzymes activity in response to fat overload [38]. In fact, both obesity and high fat diets are associated with increased protein expression of transcriptional coactivator peroxisome proliferator-activated receptor gamma coactivator 1-alpha (PGC-1α) [39], the main transcription factor regulating mitochondrial biogenesis and oxidative phosphorylation enzymes expression [40]. However, we found no associations between IMCL and mitochondrial enzymes activity. In this group of obese patients, insulin resistance appears to be associated with higher complex I activity, which is not the case with diabetes mellitus. Also, insulin resistance negatively correlated with complex IV activity. Mitochondrial dysfunction may not be a cause, but rather a consequence of insulin resistance. In accordance, in a chronological model of diet induced obesity in rats, mitochondrial oxidative capacity first presented a compensatory increase, whereas mitochondrial dysfunction was a late event, only appearing after changes in lipid metabolism and insulin sensitivity had occurred [41]. Regarding hepatic lesion, steatosis and fibrosis severity were progressively associated with higher complex I activity, which may suggest a muscle compensatory mechanism to a higher fat overload. Although NASH was not associated with mitochondrial enzymes activity changes, aminotransferases levels were correlated negatively with complex IV activity, which may be translated into a higher hepatic necroinflammatory activity in patients with sustained metabolic disturbance.

Regarding the insulin signaling cascade, activation of the first steps, namely insulin receptor and IRS-1, correlated with BMI, glucose levels and plasma lipids disturbance. However, in the following step, Akt phosphorylation rather presented a negative correlation with the above mentioned metabolic factors. Similar correlations were found, regarding leptin and ghrelin levels, mitochondrial enzymes activities (complex I and III), IMCL accumulation, and degree of hepatic steatosis, inflammation and fibrosis. These data suggest a more intense metabolic disturbance, with compensatory hyperinsulinism and insulin receptor stimulation in the muscle during NAFLD progression in obese patients. Also, in this set, insulin resistance in muscle seems to occur distally in insulin signaling cascade. This is corroborated by the data of Cusi et al. that described muscle resistance in the phosphoinositide-3-kinase (Pi3K)/Akt signaling pathway, with an intact stimulation of mitogen-activated protein kinase (MAPK) translating into normal insulin receptor function in obese individuals [42]. In fact, skeletal muscle ceramide content, which is increased in obese patients [43], is known to inhibit insulin signaling via inhibition at Akt levels [44]. Strong evidence suggests that muscle accumulation of diacylglycerol and long chain acyl-CoA leads to insulin resistance through the activation of serine protein kinase C, which impairs insulin receptor and IRS-1 tyrosine phosphorylation [45], [46]. In fact, muscle diacylglycerol accumulation seems to be more related with an increased supply of fatty acids in the diet, while ceramide accumulation is associated with physical inactivity [47]. Interestingly, physical inactivity may be a major factor contributing to the development of insulin resistance in morbid obesity, where an increase in diacylglycerol content may not occur [43]. However, previous studies from our group [48] showed a decrease in insulin receptor and IRS-1 tyrosine phosphorylation in morbid obese patients with severe NASH, which suggests that at some point in the progression of NAFLD, a continuous overstimulation of insulin receptor may lead to impairment in insulin signaling more proximally in the cascade.

A possible link between increased lipid IMCL and disturbances in insulin pathway signaling could be tumor necrosis factor-alpha (TNF-α). Recently, it has been shown that local generation of TNF-α in skeletal muscle acts paracrinally or autocrinally, inducing insulin resistance [49]. Locally produced pro-TNF-α is a membrane-bound protein that is then processed and released from the cell surface by the action of TNF-α converting enzyme (TACE). Tissue inhibitor of matrix metalloproteinase 3 (TIMP3) is TACE's natural inhibitor [49]. *In vitro* studies, as well as *in vivo*, in mice and humans, had shown that fatty acids and high fat diet inhibits TIMP3 expression leading to an increase in TACE activity, in the muscle and in the liver [49], [50]. In the muscle, TACE/TIMP3 deregulation associates with insulin resistance [49], and in the liver with NASH development [50], [51].

One unexpected finding was the lack of association between adiponectin levels and presence of NASH or lipid accumulation in the liver. It is produced mainly by adipocytes, however it is inversely proportional to the adipose mass and visceral obesity, most likely through a negative loop from down-regulation by tumor necrosis factor-α (TNF-α) derived from adipose tissue infiltrating macrophages. Adiponectin has favorable metabolic effects, acting as an insulin sensitizer as well as an anti-inflammatory and TNF-α antagonist [52]. It is known to decrease hepatic fat content in animal models [53] and it is believed to inversely relate with NAFLD in human; however, not all studies are consensual and a meta-analysis using liver biopsy data failed to demonstrate it [54]. Also, differences in adiponectin levels between patients with simple steatosis and NASH are still not clear [55]. The failure to show an association between adiponectin and hepatic or muscle histology, as well as with deregulation in insulin signaling may be due to lack of power to detect them. However, we can speculate that in a population of morbid obese patients, where adiponectin levels are already extremely low and associated with NAFLD, the correlation with more severe disease such as NASH is weakened.

Our study has some limitations, mainly in respect to the normal control group. When evaluating muscle morphology and muscle mitochondrial function, anthropometric, laboratorial and liver biopsy data were not collected. Also, when assessing muscle insulin cascade signaling, a comparison with a healthy lean control group was not possible. However, the main goal of the study was to assess the muscle morphology and metabolism according to liver injury in a homogenous group of patients, with similar metabolic risk factors, namely BMI.

In conclusion, obesity is associated with IMCL in a similar and parallel fashion with NAFLD. However, in opposite to the liver in which ectopic fat seems to be associated with a risk of liver disease progression with necroinflammatory and fibrogenic responses, in the muscle, ectopic fat does not lead to inflammation or myocyte necrosis. Moreover, IMCL is associated with a higher risk of NASH and advanced fibrosis. Also, muscle mitochondrial dysfunction does not appear to be a major driving mechanism to IMCL accumulation, insulin resistance and liver disease in obese patients. Skeletal muscle insulin resistance occurs at a late point in the insulin signaling cascade and is associated with IMCL and NAFLD severity.
